# Correlation of Caries Prevalence, Oral Health Behavior and Sweets Nutritional Habits among 10 to 19-Year-Old Cluj-Napoca Romanian Adolescents

**DOI:** 10.3390/ijerph17186923

**Published:** 2020-09-22

**Authors:** Cosmin Tudoroniu, Monica Popa, Simona Maria Iacob, Anca Lucia Pop, Bogdana Adriana Năsui

**Affiliations:** 1 Department of Community Health, “Iuliu Hațieganu” University of Medicine and Pharmacy, 6 Louis Pasteur Street, 400349 Cluj-Napoca, Romania; contact@dentist-tudoroniu.ro (C.T.); monica.popa@umfcluj.ro (M.P.); or adriana.nasui@umfcluj.ro (B.A.N.); 2 Department of Prosthetic Dentistry, “Iuliu Hațieganu” University of Medicine and Pharmacy Cluj-Napoca, 4 Louis Pasteur Street, 400349 Cluj-Napoca, Romania; Iacob.Simona@umfcluj.ro; 3 Department of Clinical Laboratory, Food Safety, “Carol Davila” University of Medicine and Pharmacy, 6 Traian Vuia Street, 020945 Bucharest, Romania

**Keywords:** dental health, adolescents, diet, oral health behaviors, DMFT

## Abstract

*Background and objectives*: The primary oral disease during adolescence is dental caries. Less is known about the caries prevalence, oral health behavior, and sweets nutritional habits in Romanian adolescents. The objective of this study was to assess the actual caries prevalence among Romanian adolescents in a representative area of Romania, Cluj, and to correlate with oral hygiene behaviors and dietary sugary foods intake. *Materials and methods*: We have done a cross-sectional study of 650 adolescents aged 10 to 19-years-old (average age 15.3 ± 2.8). We performed the oral dental examination according to the WHO methodology, calculated the number of decayed, missing (due to caries), and filled teeth (DMFT index), assessed the oral hygiene and dietary behaviors using a two-section valid questionnaire and statistically analyzed the interrelation between DMFT, oral hygiene and eating behaviors by multivariate statistical analysis. *Results*: (a) The caries prevalence in the adolescent population enrolled in the study was 95.5%; (b) the mean DMFT was 3.13 ± 2.0, without significant differences between the urban and rural adolescents (*p* = 0.253); lower in females than males (*p* < 0.050), (c) more than one third (33.7%, *n* = 219) of teenagers are seldom or never brush their teeth in the evening; (c) 40.6% of adolescents are missing the regular annual dental check-ups leading to an increased DMFT as shown in the multivariate analysis (*p* = 0.038); and (d) there is an increased prevalence of caries with age (*p* = 0.020), and with sugary sweetened beverages consumption (*p* = 0.028). *Conclusions*: Our study evidenced a persistent high caries prevalence in Romanian teenagers. Their dietary habits and irregular dental check-up were associated with the occurrence of dental conditions.

## 1. Introduction

In childhood, dental caries is the leading oral disease. Although preventable, dental caries still affects many children, particularly those from more disadvantaged social backgrounds. In high and middle-income countries, caries rates overall have declined in recent decades. Even so, and despite being largely preventable, the disease remains a significant public health problem in developed and developing countries because of the increase in consumption of sugary substances, poor oral hygiene practices, and inadequate use of dental services [1].

Dental caries is a multifactorial disease involving interactions between socioeconomic factors (income, organization of primary prevention), behavioral factors (quality and amount of nutrition, dental hygiene, behavioral patterns or lifestyles linked to society and culture), genetic factors (co-existing somatic disorders, proteins related to the antimicrobial activity (beta-defensin one and lysozyme-like protein), pH control (carbonic anhydrase VI), and bacterial colonization/adhesion (Lacto transferrin, mucin, and proline-rich protein Db), microbiological factors [2], and bio-geo-chemical features of the environment [3,4]. 

Primary prevention of dental caries must focus on dietary and behavioral risk factors, the most potential modifiable risk factors. Intake of nutritional sugars is the most critical risk factor for dental caries [5]. After being hydrolyzed by salivary amylase, the sugars and other fermentable carbohydrates provide the substrate for oral bacteria, which in turn lower plaque and salivary pH. The resultant effect is the beginning of tooth demineralization [6]. Since the introduction of fluoride, the worldwide incidence of dental caries has decreased, despite increases in sugar consumption [7].

Dental caries measurement includes the sum of decayed, missing, and filled teeth (DMFT index) (WHO 2000). WHO and Federation Dentaire Internationale (FDI) established the first global oral health goal: by the year 2000, children reaching the age of 12 will not possess an average of more than three decayed, missing, and filled permanent teeth (DMFT score 3) [8,9]. Most high-income countries reached or even exceeded these goals, while for many low-income countries, this remains a remote aspiration and is a significant public health problem [8].

In Romania during the past 30 years, the access of the population to dental services increased. Dental health education is provided at the beginning of every school year by the dentists that are working in school dental offices. Not all schools have an active dental health office. The school curricula also include free health education classes that teach children about healthy lifestyles and different health-related issues. Nevertheless, the Romanian model, as part of the Eastern European model, is mainly characterized by private service, focusing on curative treatments instead of oral prevention and promotion strategies, showing an insufficient prevention program due to an institutional gap between the school system and the dental and medical systems.

Comparative studies reveal that in Romanian teenagers, tooth extraction is more frequent, they visit the dentist less often, and brush their teeth less frequently than teenagers from two other EU countries (Portuguese and Swedish), as well as floss less, have a higher frequency consumption of sugary foods, and are in need for better dental care and education [10,11]; 

Surprisingly, a decrease of the DMFT was reported for Romania in 2000 (around three) for the twelve-year-old children [12], probably due to the increase of living standards, mainly for the people from urban and metropolitan areas; less is known about the 10–19-year-old adolescents oral health status during the last 20 years.

We aimed to assess at this point the caries prevalence in a significant sample of randomly selected Romanian adolescents from an administrative cluster representing the fourth biggest county in the country, Cluj [INSSE, 2016], the capital in the Northwest Romanian development region (equivalent to NUTS-II regions in the European Union) used by the European Union and the Romanian Government for statistical analysis and regional development [EU, 2016], one of the most important academic, cultural, industrial and business centers in Romania, the capital to the historical province of Transylvania. 

The study was done by clinical evaluation using the decayed, missing, and filled teeth index (DMFT), and by correlating the score to dental hygiene behaviors and diet habits. Secondarily we took into consideration potential correlations of the score with gender, urban and rural areas, and parents’ educational level. 

## 2. Materials and Methods

### 2.1. Study Design and Population

We performed a cross-sectional study based on caries examination and structured questionnaires, conducted among Romanian adolescents (males and females) aged 10 to 19-years-old (as defined by the WHO standards, Ferguson, 1993), during four months in 2017.

**Figure 1:**
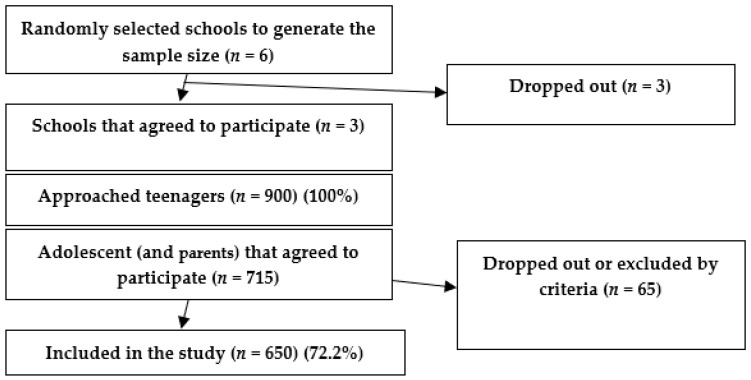
Reporting flow diagram (guideline).

We used a multistage cluster sampling method to draw a representative sample of 650 adolescents. We developed the study in the fourth biggest county in the country, Cluj, divided into smaller urban and rural administrative units (clusters). In the first stage of the study, from a total of the 167 schools in Cluj (based on data from the education management information system of the Centre of Information Technologies in Education), we generated six random numbers between 1 and 167 (i.e., the total number of schools in Cluj). We selected six schools, of which three agreed to participate in the study (a block). Of these, we randomly selected and approached 900 adolescents (300 per school from March to June 2017), from which 650 agreed to complete the study (Figure 1). The subjects were asked to complete the structured questionnaire during the dental screening sessions in the given period. 

We calculated the sample size using Paniott’s formula with an error of 5% based on the 10–19-year-old population; this amounted to 63,794, according to the Romanian Statistics Institute. We calculated a computed minimal size of 659 participants for a 99% confidence level, with 5% margin of error. Considering a response rate of about 75%, we invited in the study 900 participants.

Paniott’s formula

*n* = 1/(∆^2^ + 1/N) (1)

∆—sample margin of error, N—the total number.

We collected data from 650 adolescents assisted by parents, which met the inclusion criteria and voluntary agreement to participate in the study, informed about the aim and procedure. 

Inclusion criteria: we included in the study adolescents who at least once received dental treatment or check-ups at the dental school office, who are in good general health condition, without orthodontic treatment in progress, and without any other systemic pathology. The exclusion criteria were subjects with a history of orthognathic surgery, or a history of cervicofacial trauma, and acute or chronically orofacial muscle pain. 

### 2.2. Data Collection

We performed a two steps evaluation consisting of an oral clinical evaluation and questionnaire with two sections. We informed the students and parents that the survey was confidential and that participation was voluntary; the adolescents assisted by parents or legal guardians filled in the survey questionnaire; parents or legal guardians signed the written informed consent. To ensure confidentiality, we assigned a numerical code for each participant. The research team received the approval of the school directorate.

Ethical approval: the Research Medical Ethics Committee of the “Iuliu Hațieganu” University of Medicine and Pharmacy Cluj-Napoca approved the survey protocol (No. 287/19.06.2017).

(1) The oral clinical examination was performed by a single dentist examiner that underwent a calibration procedure following the WHO guidelines in order to have a uniform understanding and application of the International Caries Detection and Assessment System. Duplicate examinations were performed on 10% of the evaluated adolescents (*n* = 65). The kappa statistic was used to assess intra-examiner reproducibility. A trained dental nurse recorded the results of the clinical examinations performed by the dentist.
(2) The research team applied a structured original questionnaire to the participants in the study, consisting of two subsections: (a) a dental behavior section and (b) a validated food frequency questionnaire (FFQ). The dental behavior section comprised questions used in previous studies [13]. The FFQ is part of a valid questionnaire adapted to Romanian habits (EPIC Norfolk) [14]. The questionnaire was pretested and re-tested on a sample of 30 subjects. Spearman’s correlation coefficient was used to assess the reliability (*r* = 0.763). The time necessary to fill in the survey questionnaire was 25 min.
  (a) The dental behavior questionnaire section investigated: (1) the tooth brushing frequency, (2) evening tooth brushing, (3) dental visit frequency, and (4) external fluoridation (use of fluoridated toothpaste), as different frequencies (Table 1).
  (b) In the FFQ section, we assessed the food and beverages intake habits as different frequencies: never or less than 1/month, 1–3 times/month, once/week, 2–4 times/week, 5–6 times /week, once/day, 2–3 times/day, 4–5 times/day, more than six times/day. We estimated the type of different sugary foods as mentioned in the EPIC food frequency questionnaire sweets and snacks category that, for simplicity, we consolidated the categories as follows: (a) Chocolate (chocolates, single or squares, chocolate snack bars, e.g., Mars, Crunchy, cocoa, hot chocolate); (b) cakes, ice-cream, biscuits (sweet biscuits, chocolate, e.g., digestive plain, e.g., Nice, ginger, cakes, e.g., fruit, sponge, home baked/ready-made, buns, pastries, e.g., scores, lap jacks, croissants, doughnuts, fruit pies, tarts, crumbles, sponge puddings, home baked or ready-made, milk puddings, e.g., rice, custard, trifle, ice cream, choc ices); (c) sugar and candies (sweets, toffees, mints); (d) jams and syrups (jam, marmalade, honey, syrups); (e) sugary sweetened beverages (SSB’s) respectively sodas (fizzy soft drinks, e.g., Coca Cola, Sprite, Fanta, etc., lemonade); (f) added sugar (to tea, coffee, to peanuts or other nuts). In order to perform the multivariate linear regression analyses, we stratified the results into two categories—adolescents who never or less frequently, consumed these sweets and frequently.

### 2.3. Statistics

We calculated the decayed, missing, and filled permanent teeth index (DMFT/dmft) consisting of the sample’s total average number of tooth surfaces decayed, lost due to caries, and filled teeth; we did not include the third molars in the index. We correlated the DMFT indicator with the hygiene behaviors: (1) the tooth brushing frequency, (2) evening tooth brushing, (3) dental visit frequency, and (4) external fluoridation.

We performed linear regression analyses by ordinary least squares (OLS) to determine the predictors of the DMFT index: age, gender, residence, dental hygiene behaviors, and sweet food consumption. The tested model explained 17% of the variance of DMFT (F = 1, 105, *p* = 0.04). We performed the statistical analysis using the IBM Statistical Package for Social Sciences version 20 (SPSS Inc., Chicago, IL, USA) and Excel (Microsoft Office 2010, Albuquerque, NM, USA). We did the randomization using the Random Number Generators function in SPSS. We described the continuous variables using the mean and standard deviation with a 95% CI. To compare proportions and the interdependence of qualitative characteristics, we used the Chi-square test. In the case of a normal distribution of the variables, we used a t-test, and in the case of non-uniform distribution of a variable, we used the nonparametric Mann–Whitney U test. Results with *p* < 0.05 were considered statistically significant. 

## 3. Results

### 3.1. Demographics of the Studied Group

The study included 504 females and 146 males (*n* = 650). The mean age of the children was 15.3 ± 2.8 years. Males had a mean age of 13.71 ± 3.49 years, compared to female children who had a mean age of 15.8 ± 2.4 (*p* < 0.001). Total sample weight was 98 (*n* = 63,794); at a confidence level (margin of error) of 3.8% (desired to be between 5–10%). For the boys (*n* = 146), the sample weight was 223 (*n* = 32,633), and the margin of error was 8.1%. For the girls’ group (*n* = 31,161), the sample weight was 62, and the margin of error was 4.3%. 

**Figure 2:**
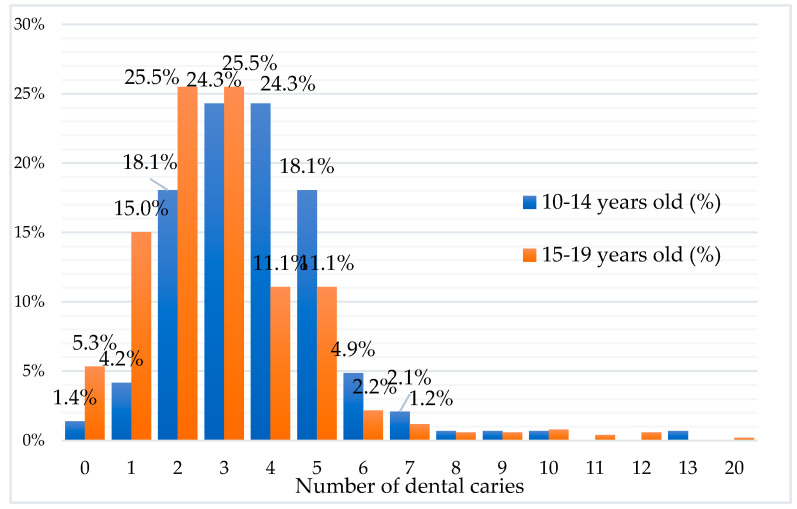
Percentages of adolescents with decayed, missing, or filled teeth by age sub-group and the number of caries.

DMFT score had values between 0 and 20 (Figure 2), median 3, with an average of 3.1 ± 2.0, and it was higher in males than females (3.4 ± 1.7 vs. 3.05 ± 2.2, *t*-test *p* = 0.030). In rural areas, the DMFT index was higher than in urban areas (3.2 ± 2.3 versus 3.09 ± 1.9, *p* = 0.280).

### 3.2. Caries Prevalence

The overall prevalence of the dental caries of different age groups in the studied group (DMFT index > zero) was 95.5% without significant differences between the urban pupils (95.9%) and rural pupils (94.8%) (*p* = 0.253). The prevalence of dental caries in the boys’ group was 95.9%, in the girls’ group was 95.2%, without statistical significance (*p* = 0.359). 

### 3.3. Dental Hygiene Behavior

The vast majority of the adolescents reported twice daily brushing (78.8%, *n* = 507) and evening tooth brushing (66.3%, *n* = 431), but still, more than one third (33.7%, *n* = 219) of teenagers are rarely or not at all brushing their teeth in the evening (39.1% in the male teenage sample and 32.2% in the female sample). A high percentage of Romanian adolescents in the present study (40.6%, *n* = 264) missed the regular dental yearly check-up in the present study, attend the dentist when they are in pain; both in females and males, without significant differences (*p* = 0.420). More girls than boys used the external fluoridation by toothpaste (77.8% compared to 66.7% in boys, *p* < 0.010) (Table 1). 

We estimated the prevalence of caries using the decayed, missing, and filled teeth score (DMFT) depending on hygiene behaviors. Our results revealed differences in caries prevalence depending on hygiene behavior, but the results are not statistically significant (Table 2). The male and female adolescents had significant differences regarding teeth brushing frequency (*p* < 0.001), evening tooth brushing (*p* = 0.002), or fluoridated toothpaste use (0.010), and dentist visit frequency (*p* = 0.002). 

### 3.4. Food Behavior

Although 62.2% of the subjects were frequently consuming chocolate, we observed significant differences between males and females regarding only the sugar-sweetened beverages (SSB’s) consumption (*p* < 0.001). Male adolescents consumed SSB’s more often than females (45.2% versus 32.7%) (Table 3).

We calculated the DMFT index of frequent sugary food consumption. The results of our study evidenced a higher caries prevalence with frequent sweets consumption (Table 4).

### 3.5. Lifestyle Factors Predicting the DMFT Index

We performed the multivariate linear regression model to estimate the caries prevalence depending on eating behaviors, dental hygienic behaviors, and demographic variables. According to the results of the study, dental caries prevalence increases with the age of Romanian adolescents (*p* = 0.028). There are statistically no differences between urban or rural areas. Dietary factors and behaviors influence caries occurrence. Sodas consumption directly influences the DMFT index (*p* = 0.028). Avoiding regular dentist check-ups leads to higher caries prevalence (*p* = 0.030) (Table 5). 

## 4. Discussion

As dental issues are a leading health concern in youth, examining factors related to dental caries is essential for prevention efforts. The current study set out to assess dental caries among a Romanian adolescent population and its relation to dental hygiene behaviors and sweets consumption. Despite the decline of prevalence in developed countries, in Europe, the Baltic States, and Eastern Europe, there is still a high level of this chronic ailment in particular developing countries undergoing the nutrition transition [10]. 

WHO and FDI global goals for oral health for the year 2000 were to lower the DMFT (below 3) in children aged 12 years (Moynihan, 2016) [11]. In Romania, the DMFT index in 12-year-old children decreased from 5 in 1985 to 3.8 in 1995. The literature data shows that the European countries have a much lower caries prevalence (as DMFT index)—0.5 in Germany (2014) [15], 0.4 in Denmark (2014), 0.6 in the Netherlands (2012), 0.7 in the UK (2011), 0.7 in Spain (2014) and 0.8 in Sweden (2011) [1,16]—compared to Romania [17]. Our study shows that Romanian adolescents have a high caries prevalence (95.5%) and a DMFT index of 3.13. The literature reveals some European countries with higher cariogenic indexes (DMFT), e.g., 4.9 in Albany [18].

As the results of our study showed, caries prevalence remains relatively high in our area. Unlike previous studies, our findings surprisingly showed no significant differences in caries prevalence between urban and rural areas [11,19]. 

The improved access to dental services can explain the decline of dental disease experience among Romanian children, better oral hygiene habits, and the enhancement of preventive measures among children (using fluoride toothpaste, frequency of dental visits, tooth sealing at an early age). Still, more than one third (33.7%, *n* = 219) of teenagers are seldom or not at all brushing their teeth in the evening, and 40.6% of teenage pupils in our study missed the regular annual dental check-ups, which was perhaps because only 59% of teenagers agreed with the fact that brushing teeth can prevent tooth decay in previous research [10].

Our study evidenced the persistence of unhealthy oral health habits in Romanian adolescents: a majority visited the dentist only in the presence of a complaint, and the visits for a regular check-up were very low. Similar patterns for dentist visit frequency were present both for males and females. Other studies also revealed children’s tendency to visit the dentist only in an emergency [14,20].

Clinical trials with fluoridated toothpaste have shown that caries can be prevented by good oral hygiene practices with the use of fluoridated toothpaste [21,22]. The oral hygiene habit of using the fluoridated toothpaste was common among males and females. 

Another factor of primary prevention is appropriate oral hygiene. A professional health provider must apply the oral hygiene procedure to control plaque (such as a dentist or a dental hygienist), or the teeth must be brushed twice per day with a fluoride dentifrice [23,24]. 

Preventive measures need to address emergency dental services and public health parental coaching sessions aimed at pain relief, first aid for oral infections and restorations, and strengthening a national tooth brushing program [25,26]. 

Previous studies have demonstrated the association between oral health behaviors and socioeconomic status and level of education. Individuals with higher income and higher education brush their teeth at least twice per day and are more likely to have had a dental examination within the past year [27]. Socioeconomic factors were also associated with the use of additional methods of dental hygiene [28]. It was also observed that socioeconomic factors are considered to be a decisive risk factor for caries in school children [28,29]. However, the results of our study did not confirm these findings, possibly due to sample selection. 

Our findings revealed that Romanian adolescents are frequently eating a lot of sweetened food. Females have better knowledge and attitudes regarding eating behaviors and sugar intake [18]. Unlike our research, some studies evidenced a higher caries prevalence in females [30,31]. 

Dental caries may result from long-term high intake of soft drinks and deterioration in oral hygiene patterns [32]. As they increase the prevalence of dental caries and enamel erosion, soft drinks have been banned from schools in Britain, France, and the United States [33], and also in the Romanian schools. 

Boys tend to drink more soft drinks than girls, and adolescents of lower parental occupation status have a higher intake of soft drinks than adolescents of higher parental occupation status [34]. Parents influence soft drink consumption. Adolescents whose mother’s education was high (university) tended to consume soft drinks (cola drinks) less often than those whose mothers had a lower educational level [35]. According to the results of our study, dental caries prevalence is influenced by sweetened soft beverages (soda) consumption. These results are essential for future prevention programs to reduce dental caries occurrence in adolescents. Policies to limit the sales of sugar-sweetened beverages should be balanced by those to increase intakes of fruits, vegetables, and other essential healthful components of diet [36].

Primary caries risk reduction preventive measures include behavioral and lifestyle modification. Reducing the frequency of intake of cariogenic carbohydrates lowers the individual caries risk. 

In addition to individual dietary recommendations and education to reduce fermentable carbohydrates intake, community-based oral health programs are essential to minimize sweet consumption in children [3,4]. Limiting sugars to 5% of energy intake is beneficial to reduce the risk of dental caries [11].

Our study showed that dental caries prevalence increased with the teenager’s age; this finding is not surprising because caries is cumulative and chronic; the DMFT measures past and present caries experiences [26] and can be stabilized by secondary preventive actions [37,38].

Our study revealed a relatively high caries incidence associated with dietary sugar consumption. On the other hand, it highlighted that there is a need to improve poor behavioral attitudes regarding oral hygiene in adolescents, to reduce dental caries prevalence. Gibson and Williams stated that twice-daily tooth brushing with fluoride toothpaste might have a more significant effect on the reduction of caries in young children than the restriction of foods sweetened with sugars [7].

## 5. Limitations

The present study has limitations. One of the limitations is the method of the questionnaire for data collection, which can lead to subjective reporting. Another limitation is the selection of the sample: adolescents studying in schools, even from rural areas of Cluj, may have the same knowledge and same behaviors as children from urban areas. The data were collected from a high-income area from our country with good access to dental health services, and maybe these results are not representative of the whole country, instead of representing a developed, urbanized sample of people. Nevertheless, the overall caries prevalence was still high.

Even though previous studies showed that the teeth brushing frequency has an essential role in the prevention of dental caries, the high quality of life in the study area can explain the adolescent’s improved oral hygiene behavior, so in the applied regression model, tooth brushing may not represent a risk factor. Due to the higher quality of life, there is an increased intake of processed food, especially of sweetened beverages—a fact proven by our study, in accordance with other studies [39]. It is possible that there is a cumulative effect of the ingested sweets that were not analyzed in the present study. The favorable effect of the increased level of dental health education may be counteracted by nutritional behavior, especially sweets intake and low attendance of regular dental office check-ups. The age and gender representativeness of the study were influenced by the sampling method (random selection of the sample). Due to the large sample size, we consider our study representative of the adolescent people in our studied area, even if the sampling error biases the results. 

## 6. Strengths

Our study is the most significant done during the last ten years in our country, analyzing a large people group that investigates the dental caries prevalence and the pro cariogenic food intake. Previous studies on the youth age range published similar statistics on 290 subjects, consisting of children and teenagers of different ages (Lucaciu, IJESPH 2020) [40], and 592 teenagers (Baciu, 2015) [14]. Together with nutritional behaviors, our study revealed the still low attendance of dental and medical check-ups in adolescents in our country, with significant consequences in the caries prevalence. 

## 7. Conclusions

Despite the decline in caries prevalence during the last 35 years in our country, the present study showed a high caries prevalence in the adolescent population of 95.5%; more than one third (33.7%) of teenagers are seldom or never brushing their teeth in the evening, and 40.6% of adolescents are missing the regular annual dental check-ups leading to higher caries prevalence. The frequent consumption of sugary sweetened beverages will generate an increased prevalence of dental caries in adolescents. 

## Figures and Tables

**Table 1:** Oral hygiene behaviors of the studied group.

Dental Hygiene Variable/Frequency	Total (No./%)	Males (No./%)	Females (No./%)	*p*
Tooth brushing frequency				
Twice/day	507 (78.0%)	95 (65.1%)	412 (81.7%)	<0.001
Less than once/day or “sometimes I forgot”	143 (22.0 %)	51 (34.9%)	92 (18.3%)	
Evening tooth brushing frequency				
Every evening	431 (66.3%)	89 (60.9%)	342 (67.8%)	0.140
Rare/not at all	219 (33.7%)	57 (39.1 %)	162 (32.2%)	
Dental visit frequency				
1–2 times/year	386 (59.4%)	82 (56.2%)	304 (60.3%)	0.420
In pain/not at all	264 (40. 6%)	64 (43.8%)	200 (39.7%)	
Fluoridated toothpaste use				
With fluoride	489 (75.2%)	97 (66.4%)	392 (77.8%)	<0.001
Without fluoride	161 (24.8%)	49 (33.6%)	112 (22.2%)	

*p* < 0.05 statistically significant.

**Table 2:** Hygiene practices and decayed, missing, and filled teeth (DMFT) index.

Hygiene Behaviors Variable	DMFT (Mean ± SD)	*p*
Teeth brushing frequency		
Twice/day	3.1 ± 1.9	<0.814
Less than once/day or “sometimes I forgot”	3.2 ± 2.1	
Evening tooth brushing frequency		
Rare/never	3.2 ± 2.2	0.770
Frequent	3.1 ± 1.9	
Dental visit frequency		
1–2 times per year	3.0 ± 2.0	0.404
In pain/ not at all	3.1 ± 2.1	
Fluoridated toothpaste use		
with fluoride	3.1 ± 1.9	0.055
without fluoride	3.2 ± 2.3	

*p* < 0.05 statistically significant.

**Table 3:** Dietary behaviors of the studied sample. Use of sugary food (type and frequency).

Food Type and Frequency	Total No %	Male No %	Female No %	*p*
Chocolate				
Never/rare	246 (37.8%)	60 (41.1%)	186 (36.9%)	0.410
Frequent	404 (62.1%)	86 (58.9%)	318 (63.1%)	
Cakes, ice-cream, biscuits				
Never/rare	405 (62.3%)	87 (59.6%)	318 (63.1%)	0.500
Frequent	245 (37.7%)	59 (40.4%)	186 (36.9%)	
Sugar, candies				
Never/rare	438 (67.4%)	99 (67.8%)	339 (67.3%)	0.980
Frequent	212 (32.6%)	47 (32.2%)	165 (32.7%)	
Jams and syrup				
Never/rare	465 (71.4%)	96 (65.7%)	369 (73.2%)	0.090
Frequent	185 (28.6%)	50 (34.2%)	135 (26.7%)	
Sugary sweetened beverages (SSB’s) (sodas)				
Never/rare	419 (64.5%)	80 (54.8%)	339 (67.3%)	<0.001
Frequent	231 (35.5%)	66 (45.2%)	165 (32.7%)	
Added Sugar				
Never/rare	228 (35.1%)	62 (42.5%)	166 (32.9%)	0.170
Frequent	422 (64.9%)	84 (57.5%)	338 (67.1%)	

*p* < 0.05 was considered statistically significant.

**Table 4:** Bivariate analysis between sugary food consumption and DMFT index.

Sugary Food Consumption	DMFT Index (Mean)	±SD	*p*
Chocolate			
Frequent	3.2	±2.1	0.610
Never/rare	3.06	±1.9	
Cakes, ice-cream, biscuits			
Frequent	3.2	±1.9	0.220
Never/rare	3.1	±2.1	
Sugar candies			
Frequent	3.2	±2.0	0.164
Never/rare	3.1	±2.1	
Jams syrups			
Frequent	3.5	±2.3	0.004
Never/rare	3.0	±1.9	
Sugary sweetened beverages SSB’s (sodas)			
Frequent	3.1	±1.8	0.414
Never/rare	3.1	±2.2	
Added Sugar			
Frequent	3.1	±1.4	0.012
Never/rare	2.6	±1.7	

*p* < 0.05 statistically significant; ±SD—standard deviation.

**Table 5:** Lifestyle factors predicting the DMFT index according to linear regression.

Independent Variable	B	±Standard Error (SE)	Beta	*t*	*p*
(Constant)	4.04	0.63		6.46	0.000
Residence (urban/rural)	−0.11	0.41	−0.02	−0.26	0.790
Age	0.84	0.38	0.17	2.21	0.020
Gender	0.13	0.39	0.03	0.32	0.750
Father education	0.22	0.37	0.04	0.59	0.560
Mother education	−0.54	0.40	−0.10	−1.3	0.180
Dental behavior					
Teeth brushing frequency	−0.23	0.36	−0.04	−0.65	0.520
Dentist visit frequency	−0.30	0.35	−0.06	0.86	0.040
Toothpaste	0.05	0.37	0.01	0.15	0.880
Eating behavior					
Sodas (SSB’s)	0.91	0.41	0.17	−2.21	0.030
Cakes, biscuits, ice cream	−0.37	0.39	−0.07	−0.96	0.340
Sugar, candies	−0.48	0.43	−0.09	−1.14	0.260
Jam, syrup	0.63	0.41	0.12	1.54	0.120
Chocolate	0.63	0.38	0.12	1.66	0.100

*p* < 0.05 statistically significant; Dependent variable: DMFT: B—unstandardized coefficients; Beta—standardized coefficients.

## Abbreviations

DMFT	Decayed, Missing, and Filled teeth index
EU	European Union
FDI	Federation Dentaire Internationale
SSB’s	Sugar-sweetened beverages
WHO	World Health Organization
EPIC Norfolk	European Prospective Investigation into Cancer—Norfolk
FFQ	Food Frequency Questionnaire
